# Pulmonary endothelium-derived PD-L1 induced by the H9N2 avian influenza virus inhibits the immune response of T cells

**DOI:** 10.1186/s12985-020-01341-x

**Published:** 2020-07-06

**Authors:** Qian Zhang, Xiang Mu, Hong Dong, Ge Hu, Tao Zhang, Cheng He, Naila Siddique

**Affiliations:** 1 Department of Preventive Veterinary Medicine, College of Veterinary Medicine, China Agricultural University, Beijing, 100193 People’s Republic of China; 2 Beijing Key Laboratory of Traditional Chinese Veterinary Medicine, Beijing University of Agriculture, Beijing, 102206 People’s Republic of China; 3 National Referece Lab for Poultry Diseases, Animal Sciences Institute, National Agricultural Research Center, Islamabad, 45500 Pakistan

**Keywords:** PD-L1, H9N2 virus, Primary microvascular endothelial cells, T cell immune response

## Abstract

**Background:**

The PD-1/PD-L1 pathway is an inhibitory signaling pathway that maintains the balance between the immune response and immunotolerance, and its overactivation in cancer and viral infections inhibits T cell function. The target cells of various viruses, microvascular endothelial cells (MECs) have been shown to be key regulatory points in immune regulation and virion diffusion in vivo during infection with multiple influenza virus subtypes. Furthermore, avian influenza virus (AIV) infection can induce immunosuppression by causing imbalances in immune responses and immune organ damage. Thus, the aim of this study was to investigate whether the H9N2 virus inhibited the immune function of T cells that migrated across MECs by upregulating PD-L1 expression on MECs.

**Methods:**

The susceptibility of rat pulmonary microvascular endothelial cells (RPMECs) to the H9N2 virus was evaluated by a plaque-forming assay and immunofluorescence staining. Then, we quantified the mRNA and protein levels of PD-L1 in RPMECs induced by H9N2 virus infection using quantitative real-time PCR and flow cytometry. The interaction between the activated T cells and RPMECs infected with the H9N2 virus was revealed using a coculture system. The effect of endothelial-derived PD-L1 on T cell function was investigated by using ELISA and flow cytometry with or without a PD-L1-specific antibody.

**Results:**

Surface staining and the plaque-forming assay showed that the H9N2 virus infected and replicated in RPMECs. Both the PD-L1 mRNA level and PD-L1 protein level were upregulated in RPMECs infected with the H9N2 virus. H9N2 virus-induced PD-L1 expression significantly reduced the secretions of IL-2, IFN-γ and granzyme B and perforin expression in T cells. The above data were significantly increased after treatment with an anti-PD-L1 antibody, confirming the above mentioned findings. In addition, the induction of PD-L1 expression decreased the proliferative capacity of the cocultured T cells but did not affect the apoptosis rate of T cells.

**Conclusions:**

Taken together, the results suggest that the H9N2 virus is able to inhibit the T cell immune response by upregulating PD-L1 expression in pulmonary microvascular endothelial cells.

## Background

The avian influenza virus (AIV) H9N2 subtype is one of the major pathogens that affect poultry and was first discovered in the United States in 1966. Recent studies have shown that in addition to infecting fowl, the H9N2 virus also infects mammals such as humans and pigs, indicating cross-species transmission [1, 2]. Furthermore, the H5N6, H7N9 and H10N8 subtypes, which have evolved from the H9N2 virus through mutations in 6 different genes, cause severe respiratory symptoms in humans [3]. Due to the mild respiratory symptoms of H9N2 virus infection, adequate preventive measures have not been taken to control the spread of the virus, thereby allowing it to evolve [4, 5]. The H9N2 virus often causes secondary infection with other pathogens, due to the downregulation of the host immune response, resulting in respiratory symptoms, aggravation of vaccine failure and even death.

Programmed death ligand 1 (PD-L1), also known as B7-H1 or CD274, is expressed on the activated T cells, B cells, macrophages, tumor cells, interstitial cells, and vascular endothelial cells (ECs), PD-L1 regulates inflammation in the heart, liver, placenta, cornea, retina, and etc. [6, 7]. The abnormal activation and expression of the PD-L1/PD-1 axis plays an important role in tumor development, chronic infection, and autoimmune diseases [8]. The binding of PD-L1 to its receptor PD-1 promotes the immune escape of viruses by suppressing the immune function of T cells [9–11]. The PD-1/PD-L1 pathway blocks hepatitis B virus (HBV)-specific CD8+ T cells by targeting IL-2-mediated STAT-5 phosphorylation, which accelerates hepatitis B progression [12]. Similarly, retroviruses inhibit CD8+ T cell expansion and cytotoxicity by inducing PD-L1 overexpression [13], while respiratory syncytial virus (RSV) induces PD-L1 in bronchial ECs, which reduces the secretion of cytotoxic molecules by effector CD8+ T cells and ensures the survival of the virus in infected cells [14]. ECs are a target of multiple viruses and trigger both innate and specific immune responses by expressing specific receptors [15, 16]. Studies have shown that ECs are the key regulators of the immune response and virion diffusion during infection with multiple subtypes of the influenza virus [16, 17]. However, it is still unclear whether ECs infected by the H9N2 virus affect the T cell immune response, especially in terms of the production of antiviral and cytotoxic proteins. The aims of this study were to investigate whether the H9N2 virus infected primary pulmonary microvascular ECs (PMECs) and whether it could induce PD-L1 expression in PMECs, thereby affecting the immune function of T cells.

## Methods

### Experimental protocol

We first investigated whether the H9N2 virus infected and replicated in RPMECs using immunofluorescence staining and a plaque-forming assay. Then, we quantified PD-L1 expression in RPMECs induced by H9N2 virus infection using RT-PCR and flow cytometry. Then, the effect of the induction of PD-L1 expression on the function of T cells was observed by using a coculture system established in transwell chambers with 6-well inserts. Finally, we investigated the levels of TNF-α and IFN-γ in the culture supernatants of RPMECs infected with the H9N2 virus.

### Isolation of RPMECs and T cells

RPMECs were isolated from the lungs as previously described with some modifications [18]. Briefly, the excised lung tissues from seven-day-old specific pathogen-free (SPF) F344 rats were rolled on dry filter paper to remove the mucosal layer, washed with phosphate buffered saline (PBS), and minced in fetal bovine serum (FBS, Gibco, Carlsbad, CA, USA). The tissue mass was then seeded in a culture plate, and the excess serum was discarded. The cells that migrated from the tissue blocks were digested with trypsin, washed with PBS, and incubated with a FITC-conjugated anti-CD31 antibody (Abcam, Cambridge, UK). RPMECs were purified by flow cytometry and cultured in EC basal medium (EBM; Lonza, Basel, Switzerland) supplemented with 20% FBS (Gibco, Carlsbad, CA, USA) and 10 ng/mL VEGF165 (PeproTech, NJ, USA). The purity of the endothelial cells was verified by staining for vascular endothelial growth factor receptor 2 (VEGFr2) under observation of a laser scanning confocal microscope (Leica TCS SP5, Leica Microsystems, Wetzlar, Germany). In brief, the cells were seeded on the bottom of a glass dish and fixed with methanol-acetone (1:1) for 20 min at room temperature. After being rinsed with PBS, the cells were incubated with a polyclonal rabbit antibody against rat VEGFr2 (Abcam, Shanghai, China) at 37 °C for 45 min, washed three times with PBS. and incubated with a FITC-labeled goat anti-rabbit secondary antibody (Origene, Rockville, MD, USA) at 37 °C for 30 min. The cell nuclei were counterstained with DAPI (4,6-diamidino-2-phenylindole dihydrochloride; Cell Signaling Technology, Danvers, MA, USA).

To isolate rat T cells, blood was collected from F344 rats, and peripheral blood mononuclear cells (PBMCs) were isolated by polysucrose and sodium diatrizoate density gradient separation (Sigma Aldrich, Shanghai, China) as previously described [19]. The PBMCs were incubated in RPMI 1640 medium containing 10% FBS at 37 °C under 5% CO_2_ for 2 h to facilitate monocyte adhesion. Negative selection enrichment columns (R&D Systems, MN, USA) were then used to enrich T cells according to the manufacturer’s protocol. The isolated T cells were cultured in RPMI 1640 medium (Gibco, Carlsbad, CA, USA) supplemented with 10% FBS for 72 h, and cells isolated from the unvaccinated rats were cultured in the same medium containing 5 μg/mL CD3 (Santa Cruz Biotechnology, Dallas, USA), 5 μg/mL CD28 (Abcam, Cambridge, UK) and 10 μg/ml PHA (Sigma Aldrich, Shanghai, China) for 72 h. To activate the isolated T cells, F344 rats were infected with 100 μL virus solution (2 × 10^7^ plaque-forming units, PFUs) by nasal drip, and then T cells were isolated on the 7th day postinfection.

### In vitro virus infection

The H9N2 virus (Ck/HB/4/08) was inoculated into 9-day-old SPF chicken embryos, and virus titers were determined by measuring PFUs. Madin-Darby canine kidney (MDCK, CCL-34, ATCC) cells used for PFUs were cultured in DMEM media supplemented with 5% FBS. AIV-specific sialic acid α-2,3-galactose receptor (SA2-3Gal) expression was confirmed by biotinylated *Maackia amurensis* lectin II (VECTOR, CA, USA) staining and then followed by staining with FITC-conjugated avidin D (green) and DAPI (blue) for nuclei. To assess H9N2 virus infection, RPMECs were washed with PBS, inoculated with virus at different multiplicities of infection (MOIs) and incubated for 1 h. Then, the cells were washed with PBS and incubated with DMEM, 0.2% bovine serum albumin (Gibco, Carlsbad, CA, USA) and 0.2 μg/mL TPCK-treated trypsin [20]. Viral titers in the supernatants were measured using PFUs. To investigate the PD-L1 level induced by inactivated H9N2 virus, viral particles were inactivated using 0.094% β-propionolactone (BPL; SERVA Electrophoresis, Heidelberg, Germany) according to a previously described protocol [21].

### RPMEC/T cell coculture system

The T cell/RPMEC coculture system was established in transwell chambers with 6-well inserts (Corning, Shanghai, China). The RPMECs were seeded in the upper chambers at a concentration of 1 × 10^5^ cells/well, and the confluence of the RPMEC monolayers was detected on days 0, 1, 2 and 3 by measuring permeability to FITC-labeled dextran (Sigma Aldrich, Shanghai, China). Then, RPMECs were infected with the H9N2 virus or inoculated with viral particles. After 24 h, T cells were plated over the infected monolayer and incubated for 8 h, Afterwards, the migrated T cells in the lower chamber were harvested and analyzed further. A viral particle control was used since normal MECs expressed very low levels of adhesion molecules, which caused a decreased proportion of migrating T cells. The transmigrated T cells in samples from the bottom chamber were counted by a TC-20 cell counter (Bio-Rad, CA, USA).

### RT-PCR

RPMECs infected with live H9N2 virus or inoculated with viral particles were harvested at 6, 12 and 24 h postinfection, and total RNA was isolated using TRIzol reagent (Invitrogen, Carlsbad, CA, USA). The fold change of the PD-L1 mRNA level in different groups relative to the control group was calculated using GAPDH as the housekeeping gene. The primer sequences were as follows: GAPDH: F, 5′ ACAACTTTGGTATCGTGGAAGGAC3’ and R, 5’AGGGATGATGTTCTGGAGAGCC3’; PD-L1: F, 5’GGAGGACCTGAAGCCTCAAC3’ and R, 5’CGTCCTGCAGCTTGACATCT3’.

### Flow cytometry

The RPMECs were harvested, and a single cell suspension was obtained [22]. Then, the cells were incubated with a PE-labeled antibody against PD-L1 (BioLegend, CA, USA) and a FITC-labeled antibody against H9N2 virus hemagglutinin (Sino Biological, Beijing, China). To detect intracellular perforin, the T cells were fixed, permeabilized with 4% paraformaldehyde in PBS for 10 min at room temperature and stained with an anti-perforin primary antibody (Santa Cruz Biotechnology, Dallas, USA) and a FITC-labeled secondary antibody (ORIGENE, Rockville, MD, USA). All labeled cells were analyzed by flow cytometry, and 10,000 events were acquired per sample. The protein expression levels were evaluated in terms of the percentage of positively labeled cells.

### MTT assay

To determine the proliferation rate of the migrated T cells, T cells harvested from the coculture system were resuspended in medium and seeded in 96-well plates at a density of 1 × 10^4^ cells/well. After adding PHA to a final concentration of 5 μg/mL, the cells were cultured for 24, 48 or 72 h. Then 10 μL 3-(4, 5-Dimethyl-2-Thiazolyl)-2, 5-Diphenyl-2-H-Tetrazolium Bromide (MTT, 5 mg/mL, Sigma, Shanghai, China) solution was added to each well for 4 h, followed by 150 μL DMSO (Sigma, Shanghai, China). Finally, the optical density at 490 nm was measured at each time point.

### Annexin V-FITC and propidium iodide staining

To detect the apoptosis rate of the migrated T cells, cells were stained with Annexin V-FITC and PI for 20 min according to the manufacturer’s protocol (Santa Cruz Biotechnology, Dallas, USA). The percentage of apoptotic cells was measured by flow cytometry.

### ELISA assay

To evaluate the levels of IL-2, IFN-γ and granzyme B, T cells that migrated to the lower chamber were harvested, reseeded in a 12-well plate, and cultured for 48 h. The supernatants were collected and measured by ELISA kits (R&D Systems Inc., MN, USA) according to the manufacturer’s instructions. To determine the levels of IFN-γ and TNF-α induced by the H9N2 virus, T cells were infected with the H9N2 virus at an MOI of 1 or 5. Supernatants were collected from each group at 12 h and 24 h postinfection and analyzed using ELISA kits (R&D Systems Inc., USA).

### PD-L1 CRISPR activation plasmid transfection

For the overexpression assay, RPMECs seeded in upper chambers at a concentration of 1 × 10^5^ cells/well were transfected with the control plasmid or PD-L1 CRISPR activation plasmid (the details of the plasmids were provided in the supplementary material) using a Lipofectamine 3000 transfection reagent kit (Invitrogen, Carlsbad, CA, USA). According to the kit instructions, mixtures of plasmid (2 μg) and Lipofectamine 3000 transfection reagent (7.5 μL) using Opti-MEM were prepared. Then, the mixture was added to the RPMECs for 72 h at 37 °C in a 5% CO_2_ incubator. The overexpression level of PD-L1 was detected at 72 h after transfection by western blotting, and the ratio of PD-L1 to β-actin was determined by ImageJ software (NIH, USA).

### Statistical analysis

The data were analyzed using GraphPad Prism software 6.0 (GraphPad, La Jolla, CA, USA). The results were expressed as the mean ± standard deviation (SD) of at least 3 independent experiments. The different groups were compared using Student’s t-test or one-way analysis of variance (ANOVA) as appropriate. A *p*-value < 0.05 was considered statistically significant.

## Results

### H9N2 virus infects and proliferates in RPMECs

**Fig. 1:**
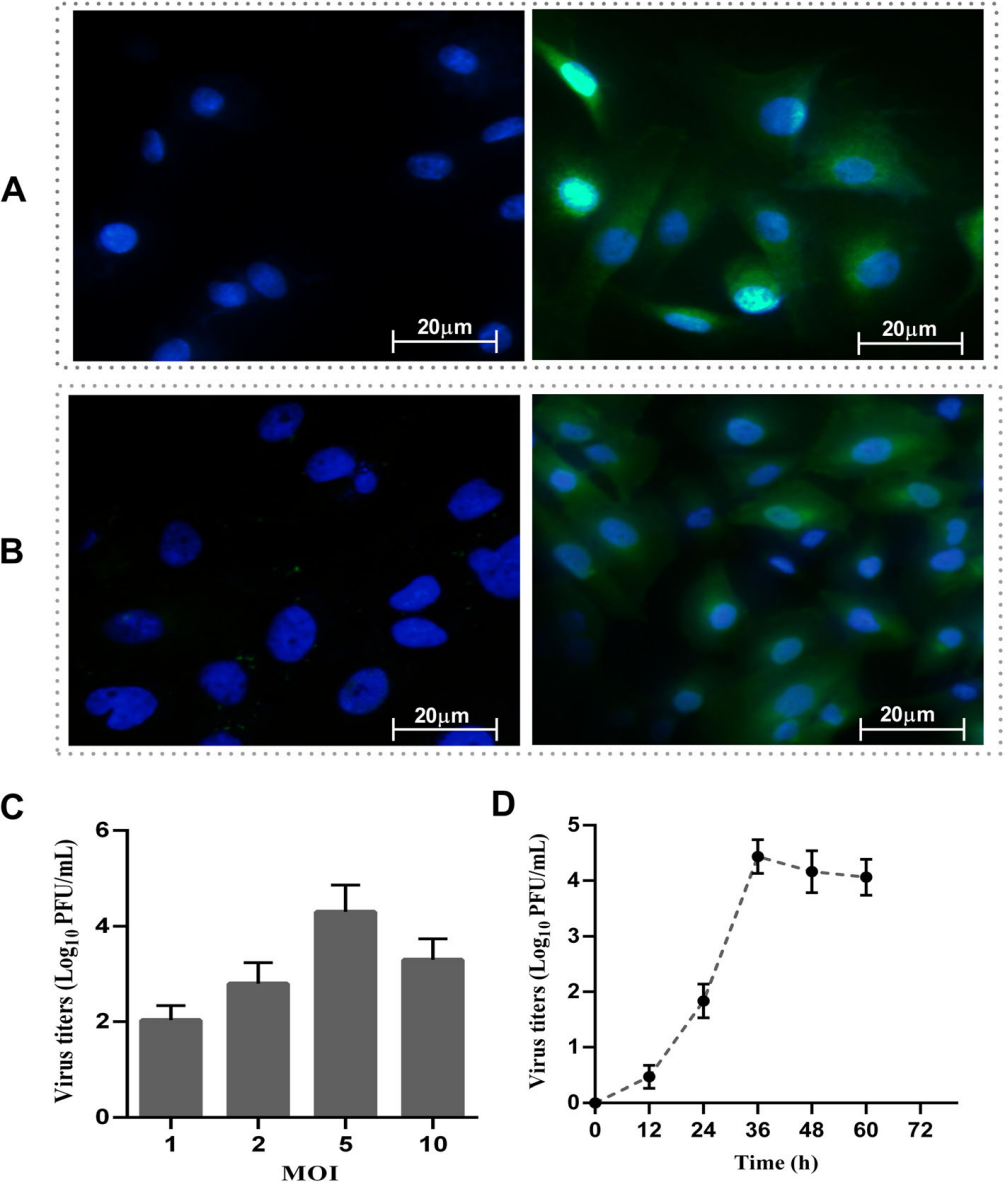
RPMECs were susceptible to H9N2 infection. **a** Expression of vascular endothelial growth factor receptor 2 (VEGFr2) in the RPMECs. The RPMECs were seeded on the bottom of a glass dish and fixed with methanol-acetone. After being rinsed with PBS, the cells were incubated with a polyclonal rabbit antibody against rat VEGFr2 and a FITC-labeled goat anti-rabbit secondary antibody (green). The cell nuclei were counterstained with DAPI (blue). The left picture was a negative control that was incubated with the secondary antibody. **b** The expression of the AIV-specific SA2-3Gal receptor on RPMECs. The cells were incubated without lectin (negative control, left picture) or with MAA II and then stained with FITC-conjugated avidin D (green) and DAPI (blue). **c** Viral titers in the supernatants at 24 h post infection with the H9N2 virus with different MOIs. **d** Viral titers at 0, 12, 24, 36 and 48 h post infection at an MOI of 1

As shown in Fig. 1 a, the expression of positive vascular endothelial growth factor receptor 2 was found in the MECs. Since AIVs preferentially bind to SA2-3Gal, we also analyzed the surface pattern of SA2-3Gal expression on RPMECs and found that the specific receptor for the H9N2 virus was expressed in the RPMECs (Fig. 1b). The plaque-forming assay showed that infection at MOIs of 1, 2, 5 and 10 produced 2.03 × 10^4^, 2.8 × 10^4^, 4.3 × 10^4^ and 3.3 × 10^4^ PFUs/ml, respectively, in the RPMECs after 24 h (Fig. 1c). Subsequently, we tested infection at an MOI of 1 for different incubation times and found that viral titers peaked at 36 h (Fig. 1d). Taken together, these results suggest that RPMECs facilitate H9N2 virus proliferation in the presence of exogenous trypsin.

### H9N2 infection upregulates PD-L1 expression in RPMECs

**Fig. 2:**
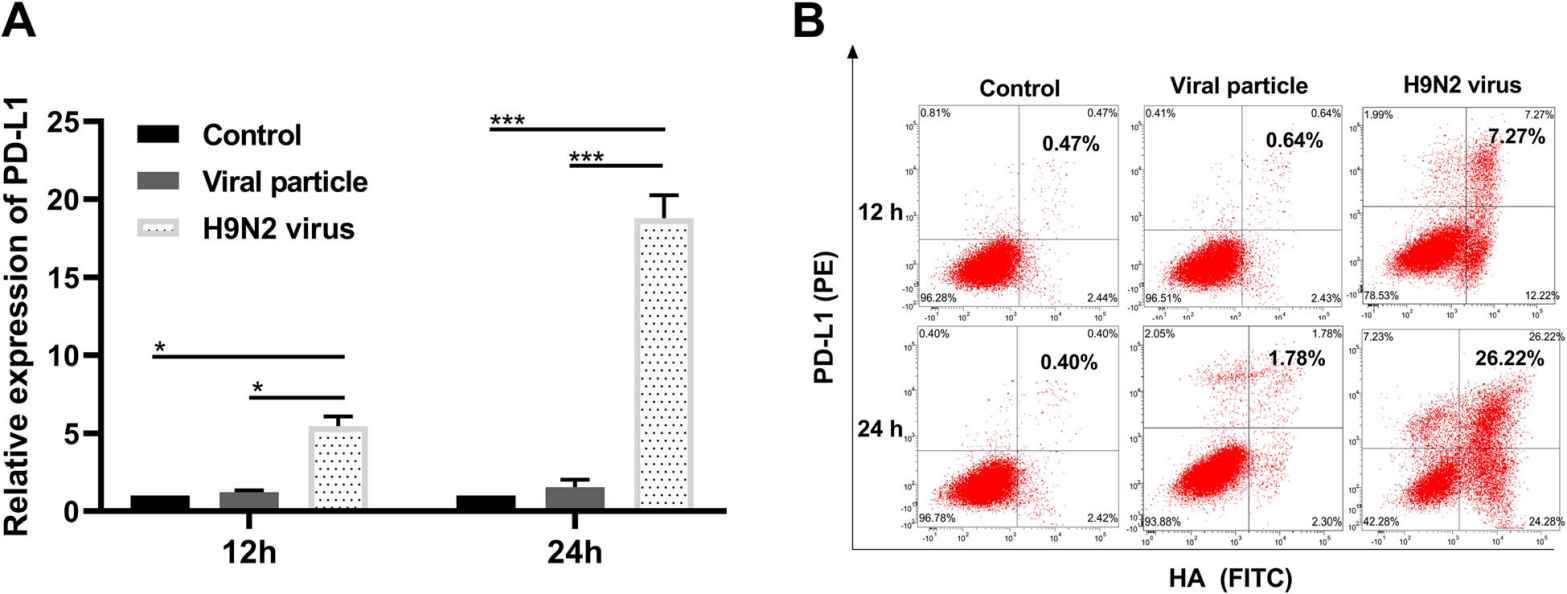
PD-L1 expression in the RPMECs infected with the live H9N2 virus or with the inactivated viral particles. PD-L1 mRNA and protein levels in the RPMECs were detected at 12 and 24 h post infection with the H9N2 virus (H9N2 virus) or with the inactivated  H9N2 viral particles at an MOI of 5 (Viral particle). **a** PD-L1 mRNA levels post infection with the live H9N2 or with the inactivated H9N2 viral particles. **b** PD-L1 protein levels post infection with the live H9N2 or with the inactivated H9N2 viral particles. * Statistical analysis among the live H9N2 group, the inactivated H9N2 viral particle group and the control group at 12 h and 24h (*P < 0.05, ***P < 0.001)

PD-L1 mRNA and protein levels in the differentially treated RPMECs were detected by RT-PCR and flow cytometry. The results showed that the inactivated viral particle did not induce the expression of PD-L1. In contrast, live H9N2 virus significantly induced the expression of PD-L1 at the mRNA and protein levels in RPMECs in a time-dependent manner (Fig. 2a, b, *P* < 0.05). The higher levels of PD-L1 at 24 h compared to 12 h postinfection were likely due to virus replication in RPMECs. Taken together, these results suggest that H9N2 virus infection significantly increases PD-L1 expression in the RPMECs.

### IFN-γ and TNF-α levels in the RPMECs infected with the H9N2 virus

**Fig. 3:**
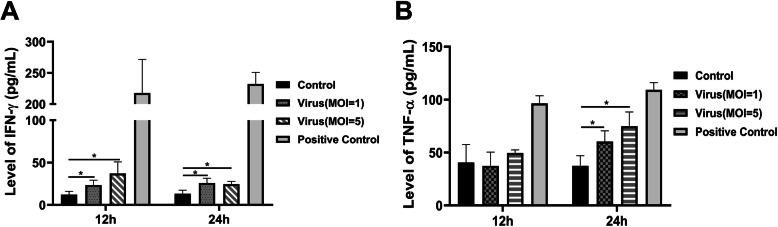
The levels of IFN-γ and TNF-α in the RPMECs. The RPMECs were infected with the live H9N2 virus at an MOI of 1 or 5, and then the supernatants were collected at 12 h and 24 h postinfection. The levels of IFN-γ and TNF-α were detected using ELISA kits according to the instructions of the ELISA kits. The values represent the means of three independent experiments plus the standard deviations. **a** IFN-γ levels in the RPMECs. **b** TNF-α levels in the RPMECs. * Statistical analysis  among the H9N2 virus group(MOI=1), the H9N2 virus group (MOI=5) and the control group (*P < 0.05, ***P < 0.001, ANOVA)

As described previously, IFN-γ can rapidly induce PD-L1 expression [23], and TNF-α can enhance the ability of IFN-γ to induce PD-L1 expression [24]. Therefore, we investigated whether IFN-γ and TNF-α were induced in the H9N2 virus-infected RPMECs. The results showed that H9N2 virus infection significantly increased the levels of IFN-γ in the RPMECs at 12 and 24 h (Fig. 3a, *P* < 0.05). However, the levels of TNF-α were only elevated at 24 h (Fig. 3b, P < 0.05). This result may explain why the level of PD-L1 at 24 h was higher than that at 12 h.

### H9N2 virus-induced PD-L1 inhibits the immune response of T cells in vitro and in vivo

**Fig. 4:**
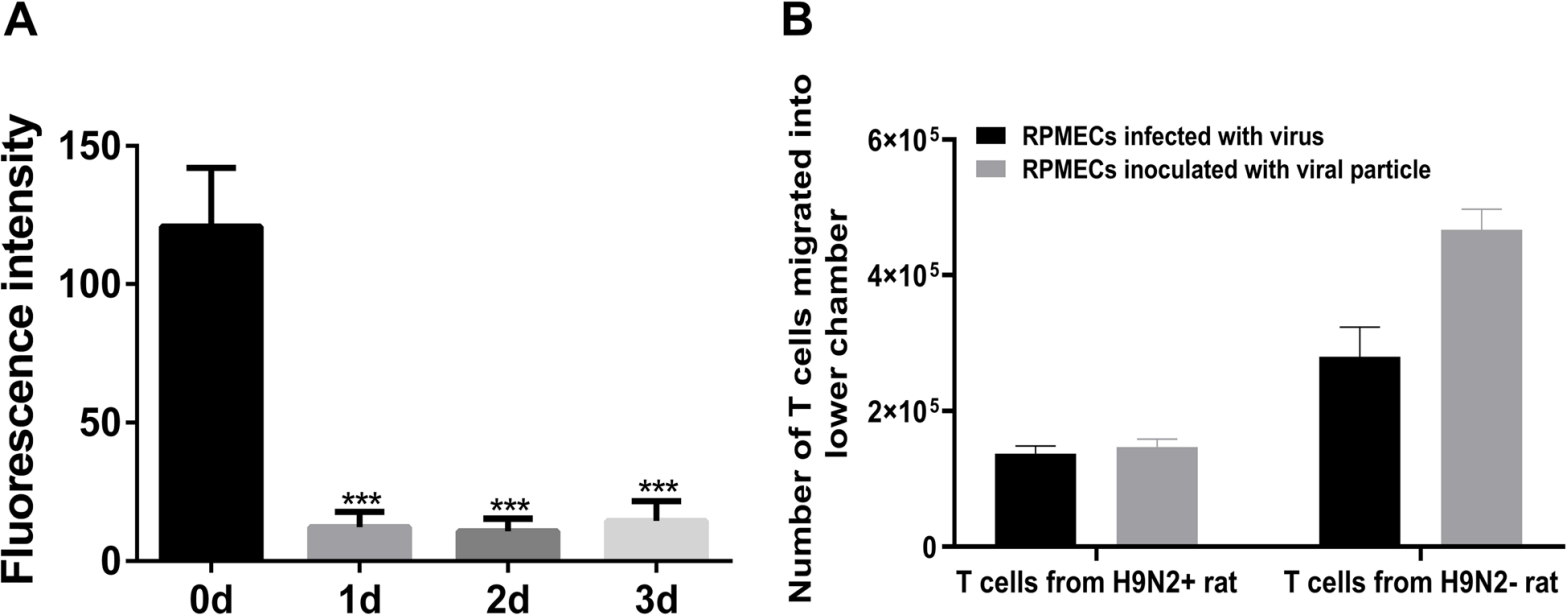
Determination of transmigrated T cells. RPMECs were cultured on transwell filters, and confluence was assessed by measuring the fluorescence intensity in the lower chamber. T cells (1 × 10^6^ cells per well) were added to the top chamber and allowed to transmigrate for 8 h. Then, the transmigrated T cells were counted by detecting fluorescence intensity. **a** The confluence of RPMECs on days 0, 1, 2 and 3. **b** The numbers of transmigrated T cells. T cells from H9N2+rat indicated that T cells were isolated from H9N2 virus-infected rats. T cells from H9N2-rat indicated that T cells were isolated from rats with the inactivated H9N2 viral particles. * Statistical analysis of RPMECs confluence at different time points (***P < 0.001, t-test)

**Fig. 5:**
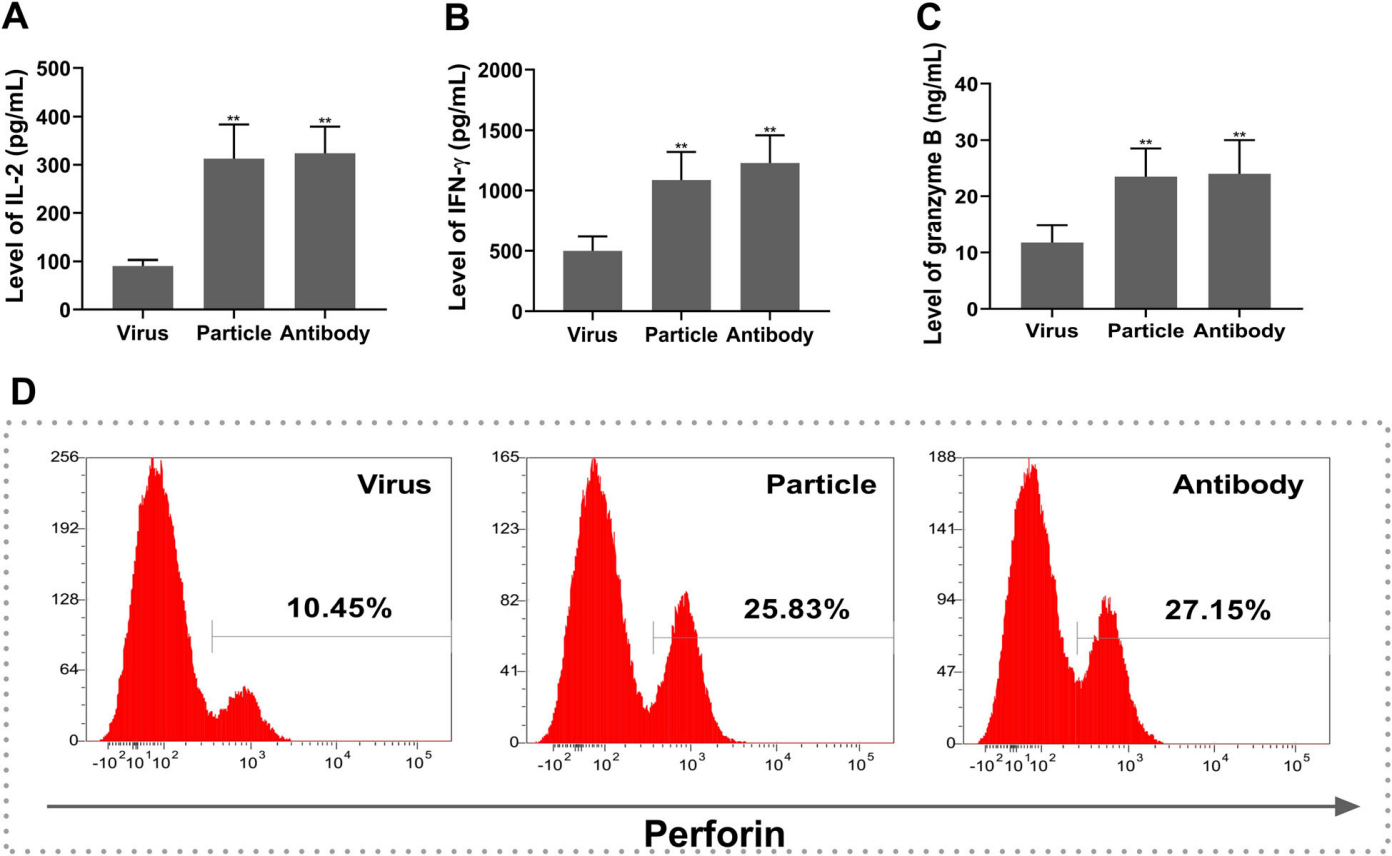
Secretions of cytotoxic factors by migrating T cells cocultured with differentially treated RPMECs. RPMECs were divided into three groups: The RPMECs were infected with the live H9N2 virus at 5 MOI(Virus), the RPMECs were inoculated with the inactivated H9N2 virus at 5 MOI ( Particle) and the RPMECs were infected with the live H9N2 virus at 5 MOI and then treated with a PD-L1 specific antibody (Antibody). Then, the activated RPMECs were added to T cells for 8 h. Transmigrated T cells in the bottom chamber were collected and cultured. The levels of IL-2, IFN-γ and granzyme B in T cell supernatants were determined by ELISA kits. Flow cytometry was used to detect the expression of perforin in T cells. The levels of secreted IL-2 (**a**), IFN-γ (**b**) and granzyme B (**c**) and the expression of perforin (**d**) were determined in T cells cocultured with RPMECs. * Statistical analysis post treatment with the H9N2 virus, the inactivated H9N2 virus particles or the PD-L1 antibody (**P < 0.01, t-test)

A transwell coculture system was used to investigate the role of H9N2 virus-induced PD-L1 expression in the interaction between RPMECs and T cells. In this experiment, we first investigated the migration rate of T cells across a RPMEC monolayer infected with the live H9N2 virus or inoculated with viral particles. As shown in Fig. 4a, RPMECs cultured on the transwell filter were completely fused; thus, the T cells could not pass through the pores of the transwell filter. The migration of T cells across the RPMEC monolayer was observed (Fig. 4b). The levels of IL-2, IFN-γ, granzyme B and perforin induced by T cells that migrated across RPMECs were evaluated by using ELISA kits and flow cytometry. The results showed that the levels of IL-2 (Fig. 5a, *P* < 0.05), IFN-γ (Fig. 5b, *P* < 0.05) and granzyme B (Fig. 5c, *P* < 0.05) secreted by T cells that migrated across RPMECs infected with the H9N2 virus were significantly decreased. In addition, the proportion of perforin+ T cells was also significantly reduced following migration across RPMECs infected with the H9N2 virus (Fig. 5d, *P* < 0.05, t-test). However, the levels of these factors expressed by T cells that migrated across RPMECs infected with the H9N2 virus were restored when we blocked PD-L1 with a specific antibody (Fig. 5a, b, and c).

**Fig. 6:**
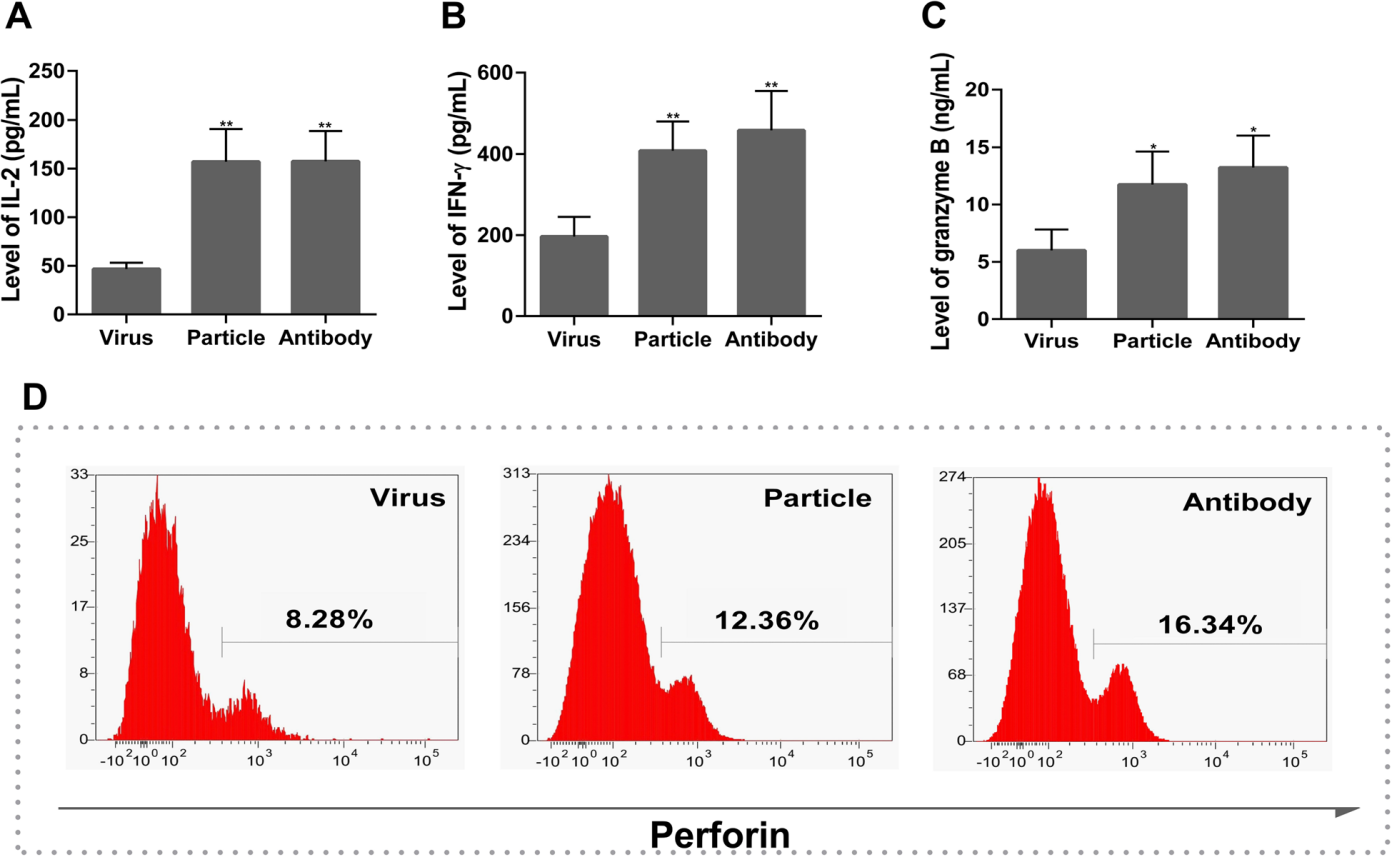
Secretion of cytotoxic factors by the migrated T cells from H9N2 virus-infected rats. The RPMECs were divided into 3 groups: RPMECs from rats infected with the live H9N2 virus (Virus), RPMECs from rats inoculated with the inactivated H9N2 virus (Particle), RPMECs from rats infected the live H9N2 virus and then treated with a PD-L1-specific antibody (Antibody). Then the activated RPMECs were added to the T for 8 h. The levels of secreted IL-2 (**a**), IFN-γ (B **b**) and granzyme B (**c**) by T cells and the expression of perforin (**d**) post the different RPMECs cocultured with T cells. * indicates the particle or antibody group compared with the virus group. Statistical analysis of cytotoxic factors post treatment with RPMECs from H9N2 virus-infected rats, or RPMECs from the viral particles or the PD-L1 antibody (*P < 0.05, **P < 0.01)

To investigate the effect of PD-L1 on the immune response of T cells from H9N2 virus-infected rats, we cocultured the T cells with RPMECs infected with the live H9N2 virus or with the viral particles. The results showed that T cells that migrated across virus-infected RPMECs expressed lower levels of IL-2 (Fig. 6a, *P* < 0.05), IFN-γ (Fig. 6b, *P* < 0.05) and granzyme B (Fig. 6c, *P* < 0.05) than T cells that migrated across viral particle-inoculated RPMECs. Similarly, the proportion of perforin+ T cells was also significantly reduced (Fig. 6d, *P* < 0.05, t-test). In the activated T cell cocultures, treatment with the anti-PD-L1 antibody restored the immune function of the T cells. All the data indicated that PD-L1 expressed on H9N2 virus-infected RPMECs inhibited the immune function of migrated T cells.

### H9N2 virus-induced PD-L1 inhibits the proliferation of T cells without affecting apoptosis

**Fig. 7:**
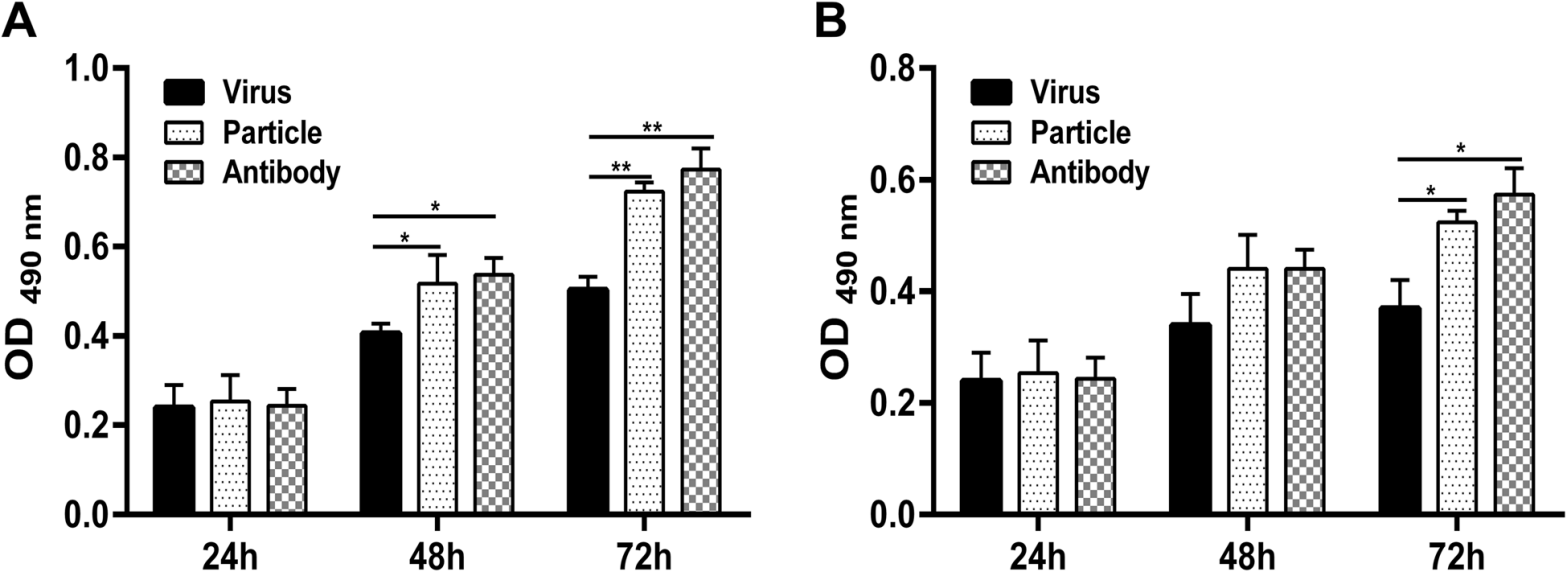
Effect of H9N2 virus-induced PD-L1 on the proliferation of T cells. RPMECs were treated with the H9N2 virus (Virus), viral particles (Particle) or the H9N2 virus plus a PD-L1 antibody (Antibody), and then the activatedT cells were added to endothelial cells for 8 h in vitro. Migrated T cells in the bottom chamber were collected for the MTT assay. The MTT results showed the proliferation rate of T cells that migrated across RPMECs. **a** The proliferation rate of the activated T cells in vitro. **b** The proliferation rate of T cells isolated from H9N2 virus-infected rats. * Statistical analysis of T cell proliferation post treated with the H9N2 virus,or the inactivated H9N2 viral particles or the H9N2 virus+ PD-L1 antibody (*P < 0.05, **P < 0.01)

**Fig. 8:**
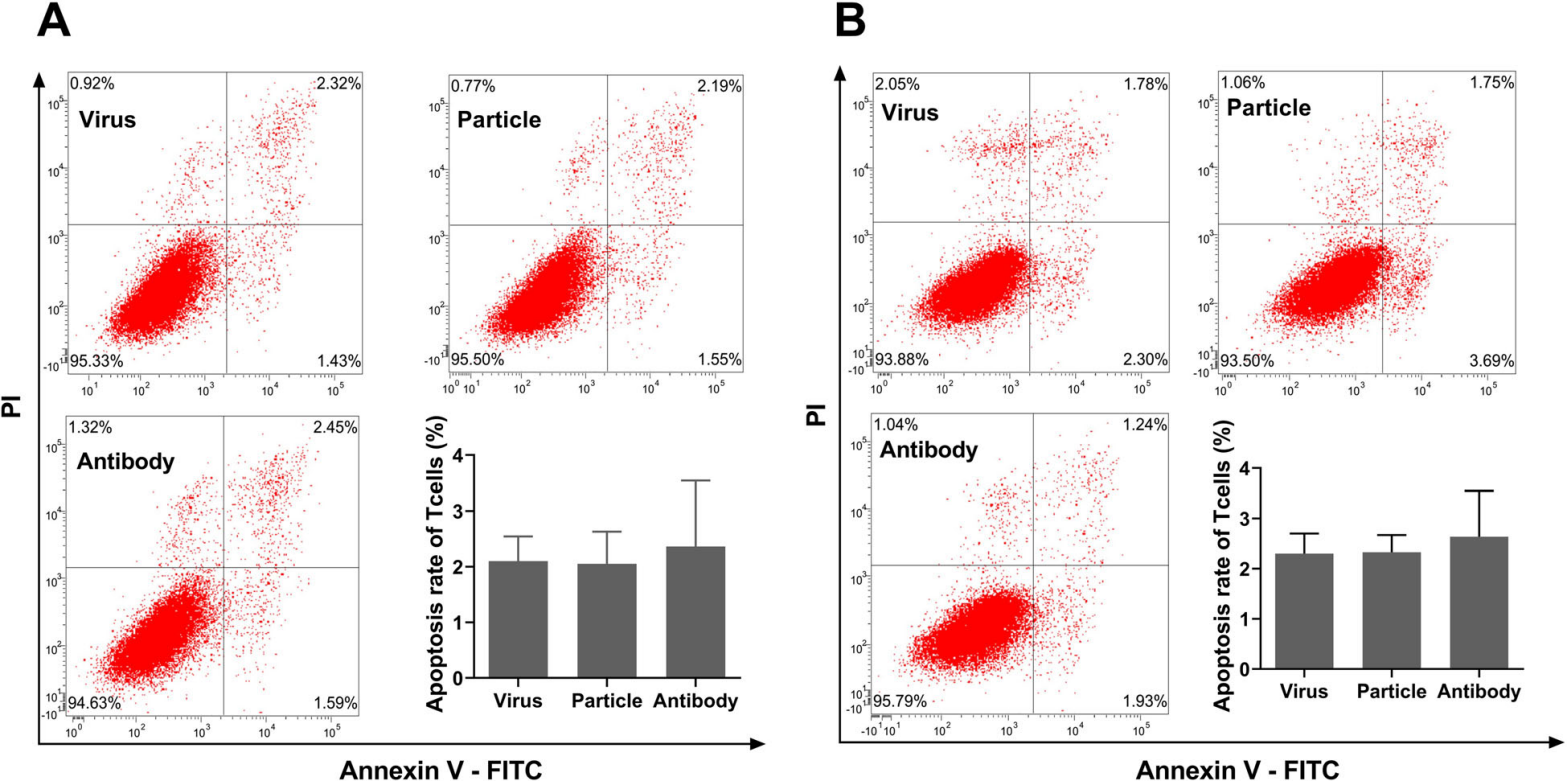
Effect of H9N2-induced PD-L1 on the apoptosis of T cells. Annexin-V/PI-stained apoptotic T cells that migrated across RPMECs treated with the H9N2 virus (Virus), viral particles (Particle) or the H9N2 virus plus a PD-L1 antibody (Antibody). **a** The apoptosis rates of T cells activated in vitro. **b** The apoptosis rates of T cells isolated from H9N2 virus-infected rats

As shown in Fig. 7a, cocultivation of the activated T cells with H9N2 virus-infected RPMECs produced a significantly lower proliferation rate than the viral particle group did, and the proliferation rate was restored after PD-L1 was blocked. Similarly, the proliferative capacity of T cells from H9N2 virus-infected rats was also suppressed after the T cells migrated across RPMECs infected with the live H9N2 virus and was restored after PD-L1 was blocked (Fig. 7). In addition, the percentages of Annexin V+ apoptotic T cells were 2.1%, 2.0% and 3.0%, respectively in the live H9N2 virus group, the viral particle group and the PD-L1 antibody group (Fig. 8a). Similar results were obtained for T cells isolated from H9N2 virus-infected rats (Fig. 8b). Taken together, these results suggested that the high levels of PD-L1 on RPMECs induced by H9N2 virus infection inhibited the proliferation of migrated T cells.

### PD-L1 overexpression significantly suppresses the immune response of T cells

**Fig. 9:**
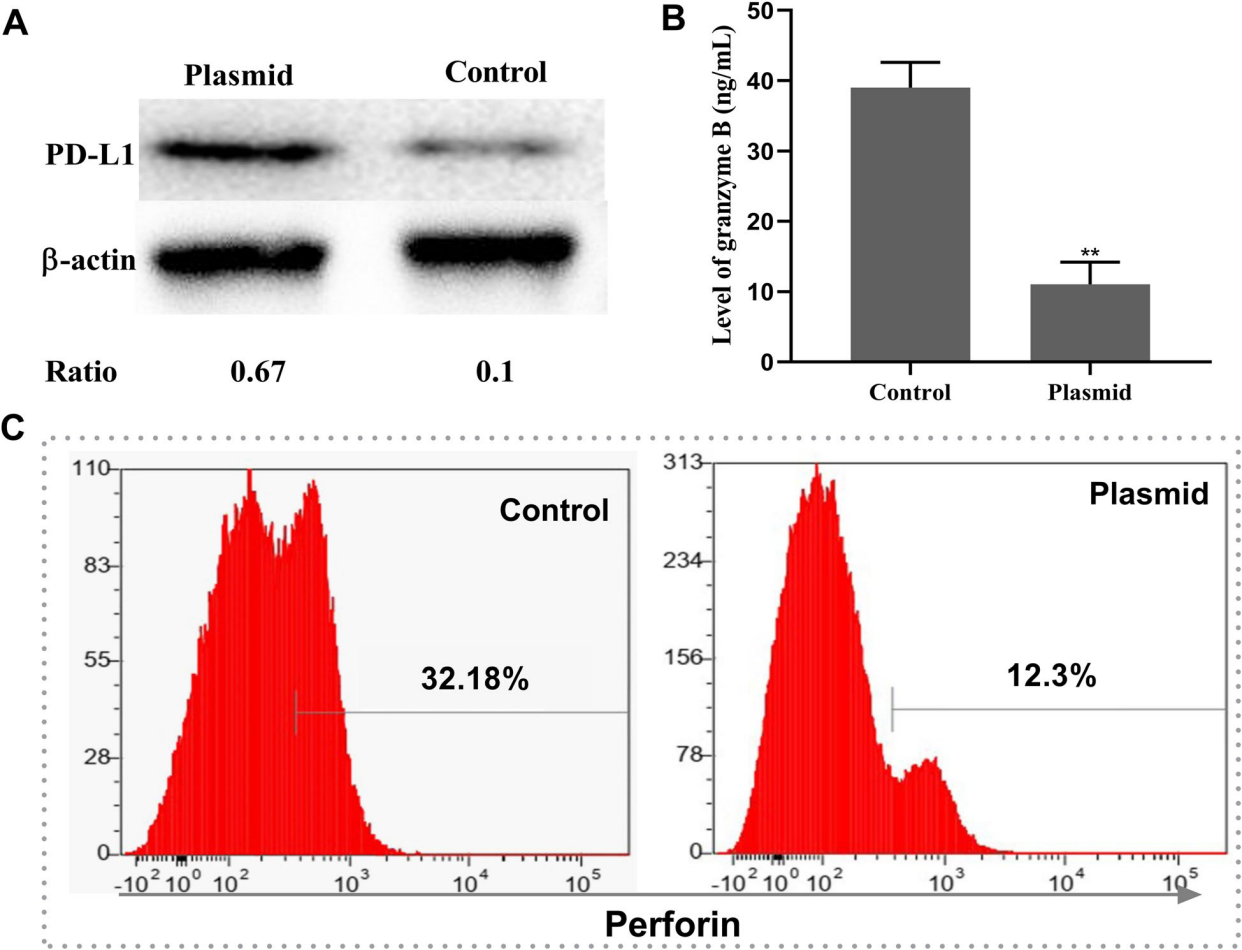
Overexpression of PD-L1 suppresses T cell function. RPMECs were transfected with control plasmid (control) or a PD-L1 CRISPR activation plasmid (plasmid) for 48 h, and then cells were inoculated with viral particles at an MOI of 5 for 24 h. Then, the activated T cells were added to the RPMECs for 8 h in vitro. Migrated T cells were collected and cultured for flow cytometry and ELISA. The overexpression of PD-L1 was detected by western blotting. **a** PD-L1 protein levels in RPMECs after plasmid transfection. **b** The levels of granzyme B in the culture supernatant of T cells. **c** The percentage of perforin-positive T cells. * indicates the plasmid group compared to the control group (**P < 0.01, t-test)

RPMECs were inoculated with viral particles for 48 h post plasmid transfection. After 24 h, the T cells were plated over an RPMEC monolayer and incubated for 8 h. Migrated T cells in the lower chamber were harvested and analyzed further. The results showed that transfection with the PD-L1 CRISPR activation plasmid elevated the expression of PD-L1 in the RPMECs (Fig. 9a). Compared to those in the control group, the levels of IL-2 and perforin were significantly decreased (Fig. 9b, and c, *P* < 0.05).

## Discussion

Currently, the H9N2 virus is widespread in poultry throughout Asia and can also infect mammals [25], including humans [26]. The efficacy of influenza virus infection depends on the presence of specific sialic acid receptors on the cell surface [27]. In general, respiratory epithelial cells are considered to be target cells of influenza viruses [28]. However, increasing evidence has shown that MECs also play an important role in the immune response to influenza virus. Moreover, the previous reports have shown that H5N1 and H7N9 subtypes can directly infect human lung MECs and replicate in EC lines [29]. Therefore, to determine the susceptibility of primary RPMECs to the H9N2 virus, we detected SA2-3Gal expression on RPMECs, which indicates that these cells are susceptible to AIV. Subsequent plaque analysis revealed that progeny virus was detected in the culture supernatant of RPMECs inoculated with the H9N2 virus, indicating the intracellular replication of the virus. The results indicate that the H9N2 virus can infect and replicate in primary RPMECs. This result may further explain the multiple organ hemorrhages post infection with H9N2 virus.

PD-L1 expression can be induced in many cell types, and increased expression has been observed in tumors and infections. A previous study showed that respiratory syncytial virus (RSV) induces PD-L1 expression on bronchial epithelial cells, which inhibits the antiviral effects of local CD8+ T cells [14], indicating that epithelial cells interact with T cells during virus infection. ECs play an important role in initiating and modulating peripheral immune responses by interacting with T cells via CD58, B7-H1, ICOS ligands, OX40 ligands and CD40 [30, 31]. MECs are considered a key regulator of immune responses to multiple influenza virus subtypes [17]. The clinicopathological changes caused by AIV are also closely related to MEC dysfunction [32]. Thus, investigating the interaction between the H9N2 virus and RPMECs helps to elucidate the immune response to H9N2 virus infection. In the present study, we demonstrated that the levels of PD-L1 are significantly upregulated in the primary RPMECs infected with the H9N2 virus. Furthermore, our results indicated that virus infection-induced PD-L1 expression transmits a negative signal to migrating T cells, resulting in the downregulation of antiviral cytokines and a decrease in cytotoxic protein production. In addition, by overexpressing PD-L1 in normal RPMECs, we found that elevated PD-L1 also inhibited the function of migrating T cells. Previous studies have shown that hepatitis C, hepatitis B and simian immunodeficiency viruses significantly increase PD-1 expression on effector T cells during the acute phase of infection before the virus becomes persistent or latent. However, the presence of PD-1 on effector T cells does not induce the depletion of these cells, indicating that the PD-1 ligand level contributes to the extent of PD-1/PD-L1 signaling during infection. Although studies have shown that PD-LI expression is elevated in T cells after viral infection, our previous microarray study indicated that PD-L1 levels were elevated in endothelial cells infected with the H9N2 virus but that the PD-1 ligand levels were not significantly increased. The ligands of PD-1 and PD-L2 are preferentially expressed on antigen-presenting cells. Therefore, we only explored the regulation of T cell function by H9N2 virus-induced PD-L1.

AIV infection escapes the host immune response either by inducing an inflammatory reaction or by inhibiting immunity via IFN-γ blockade [33], which is the primary cause of vaccine failure in livestock and poultry industry [34]. Although in vivo studies have not clearly elucidated the role of MECs during AIV infection, the lung injury seen in infected hosts indicates EC dysfunction [35]. The alveolar epithelium is separated from MECs by only a 100 nm-thick basal layer, making it easily accessible to viral progeny [36]. Furthermore, since cytokines such as IL-1, IFN-γ and TNF-α can induce PD-L1 expression, the inflammatory reaction triggered by influenza viruses may also be a factor in the induction of PD-L1 expression on ECs [37]. Moreover, our study also showed that H9N2 virus infection can increase the levels of IFN-γ and TNF-α in the primary RPMECs. In vivo studies have shown that RSV ensures its survival in infected tissue cells by inducing PD-L1 expression on bronchial epithelial cells [38]. Many viruses that cause chronic infections can evade the immune response and attenuate the antiviral T cell response via the PD-1/PD-L1 inhibitory pathway, resulting in persistent clinical signs of viral infection [39]. Similarly, the induction of PD-L1 expression on RPMECs by the H9N2 virus may be one of the mechanisms of immune escape. H9N2 virus-induced PD-L1 decreased T cell proliferation, which was restored by blocking PD-L1, but had no effect on apoptosis. Since rodent ECs express only MHC class I receptor [40], the inhibitory effect of PD-L1 on T cell proliferation is likely mediated by cell cycle arrest.

Further studies are necessary to enhance the understanding the pathogenesis provided by our present results. The exact mechanism still needs to be elucidated in further studies. In particular, we examined whether H9N2 virus infection increases PD-L1 expression in endothelial cells in vivo and also mediates immune escape.

## Conclusion

PD-L1 expression is induced in the primary RPMECs infected with the H9N2 virus, and PD-L1 induced by H9N2 virus infection inhibits antiviral interactions between RPMECs and T cells. These results indicate that PD-L1 might be a suitable target for reversing immunosuppression induced by H9N2 virus infection.

## Supplementary information

**Additional file 1: Supplementary Figure S1.** Map of the PD-L1 overexpression plasmids. According to the current genomic database of the CD274 gene. The targets were designed to include the upstream of the transcript. The PAM sequences of the target sites for CRISPR were TGG. Primers were synthesized, and the oligo dimer was inserted into the vector. DH5a competent cells were used for conversion. Plasmids A and B were mixed at a ratio of 1:1, and RPMECs were transfected with Lipofectamine 3000 reagent according to the instructions. (A) Map of plasmid 1 for MS2-P64-NSF1 expression. (B) Map of plasmid 2 for dCas9-vp64 and gRNA expression.

## Data Availability

All data generated or analyzed during this study are included in this published article.

## Abbreviations

ECs: Endothelial cells
MOI: Multiplicity of infection
RPMECs: Rat pulmonary microvascular endothelial cells
PMECs: Pulmonary microvascular ECs
VEGFr2: Vascular endothelial growth factor receptor 2
AIV: Avian influenza virus
SPF: Specific pathogen-free
HBV: Hepatitis B virus
RSV: Respiratory syncytial virus
