# Supplementary material for: Pulmonary endothelium-derived PD-L1 induced by the H9N2 avian influenza virus inhibits the immune response of T cells
Source: Virol J. 2020 Jul 6;17:92. doi: 10.1186/s12985-020-01341-x (PMC7336647; doi:10.1186/s12985-020-01341-x)
Supplement: Supplementary file 1 — Additional file 1: Supplementary Figure S1. Map of the PD-L1 overexpression plasmids. According to the current genomic database of the CD274 gene. The targets were designed to include the upstream of the transcript. The PAM sequences of the target sites for CRISPR were TGG. Primers were synthesized, and the oligo dimer was inserted into the vector. DH5a competent cells were used for conversion. Plasmids A and B were mixed at a ratio of 1:1, and RPMECs were transfected with Lipofectamine 3000 reagent according to the instructions. (A) Map of plasmid 1 for MS2-P64-NSF1 expression. (B) Map of plasmid 2 for dCas9-vp64 and gRNA expression. [file 12985_2020_1341_MOESM1_ESM.docx]

**Supplementary figure legend**

**Supplementary Fig. 1. Map of the PD-L1 overexpression plasmids.** According to the current genomic data, there is one transcription product of the CD274 gene. Based on current genomic data, targets were designed upstream of the transcript. The PAM sequences of the target sites for CRISPR were TGG. Primers were synthesized, and the oligo dimer was inserted into the vector. DH5a competent cells were used for conversion. Plasmids A and B were mixed at a ratio of 1:1, and RPMECs were transfected with Lipofectamine 3000 transfection reagent according to the instructions. (A) Map of plasmid 1, which was used for MS2-P64-NSF1 expression. (B) Map of plasmid 2, which was used for dCas9-vp64 and gRNA expression.

**Supplementary Figure 1**

A

B
